# Supplementary material for: Cellulose Isolation from Tomato Pomace Pretreated by High-Pressure Homogenization
Source: Foods. 2022 Jan 19;11(3):266. doi: 10.3390/foods11030266 (PMC8833915; doi:10.3390/foods11030266)
Supplement: Supplementary file 1 [file foods-11-00266-s001.zip › foods-1541100-supplementary.pdf]

foods

Supplementary Materials

Cellulose Isolation from Tomato Pomace Pretreated by High-Pressure Homogenization

Annachiara Pirozzi [1], Giovanna Ferrari [1,2] and Francesco Donsì [1,*]

[1] Department of Industrial Engineering, University of Salerno, Via Giovanni Paolo II, 132, 84084 Fisciano, SA, Italy; apirozzi@unisa.it (A.P.); gferrari@unisa.it (G.F.)
[2] ProdAl Scarl, University of Salerno, Via Giovanni Paolo II, 132, 84084 Fisciano, SA, Italy
* Correspondence: fdonsi@unisa.it

S1. I-optimal design

Response surface methodology (RSM) with I-Optimal Design was used for experiment planning and data processing. We have used an I-optimal design, as it minimizesthe average prediction variance across the design space [1,2]. A second-order regression model for response surface design takes the following form:

$$Y_1 = \beta_0 + \sum_{j} \beta_j X_j + \sum\sum_{i<j} \beta_{ij} X_i X_j + \varepsilon \quad (1)$$

In this equation, the values of the total phenols, used as the response variable (Y_1), were related to the experimental factors ($X_{i,j}$ with i, j = 1, 2). The coefficients of the polynomial model correspond to a constant term (β_0), linear effects (β_j), and puresecond-order interaction effects (β_{ij}), with ε being the model error.

The significant terms of the model were determined through analysis of vari- ance (ANOVA) for the response variable of a duplicate, carried out through the De-sign-Expert 11.1.2.0 software. The adequacy of the model was assessed determining the determination coefficients (R^2, Adj-R^2), and probability values (p-value).

S2. Optimization of conventional extraction

The experimental data in Figure S1 were fitted to the multilinear regression model In Equation 1, considering both the first order and second order terms, as well as the inter action effects. This graph is useful to understand the correlation between the input parameters and response variable and to enable the prediction of the optimum values of the independent variables (type of solvent and solvent concentration) that maximize the response variable (total phenols).

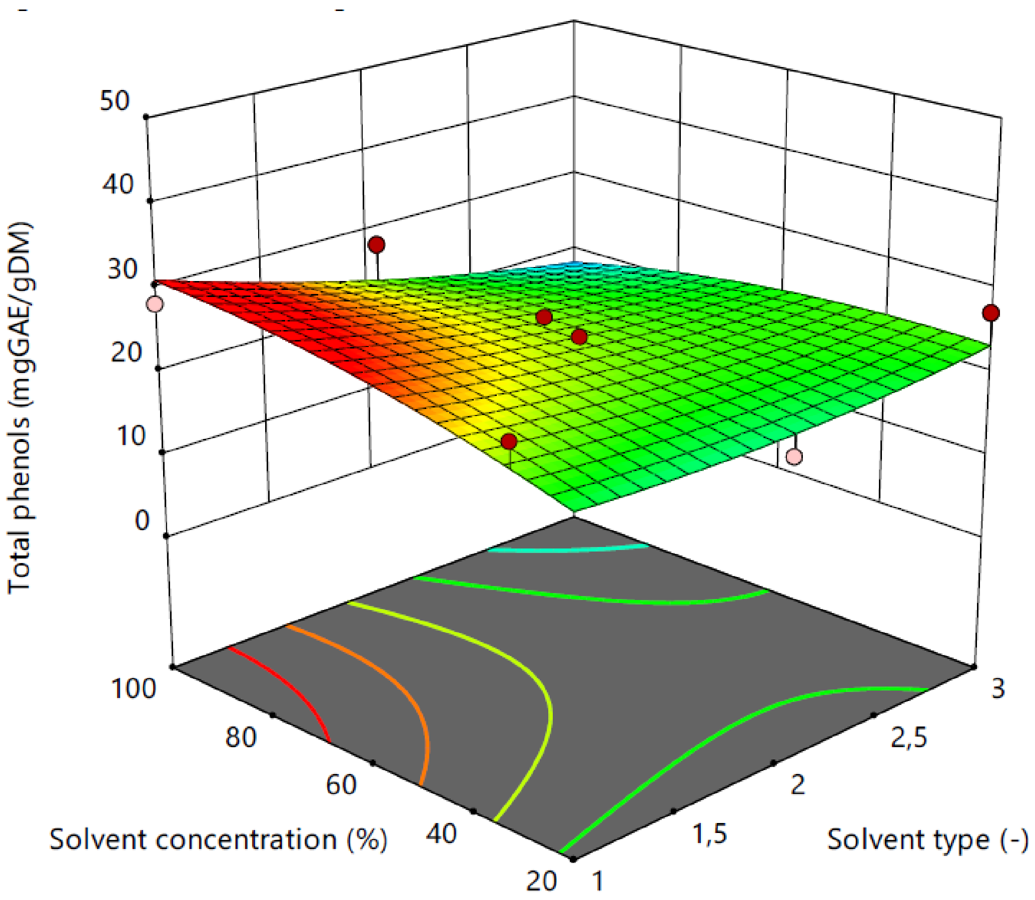

Figure S1. 3D Response surface plot presenting the synergistic effects of factors: solvent concentration and type of solvent on total phenols.

Figure S1 explicitly reports the strongly dependence of total phenols from the solvent concentration and solvent type. The total phenols decrease with the increase of solvent polarity (i.e. solvent type). Therefore, the more affine solvent, which allows maximizing the total phenols extraction, is acetone (solvent #1). In addition, the optimum concentration of acetone ranged between 70 and 100% v/v.

Table S1. ANOVA results for quadratic model fitted to total phenols extraction.

Source	Coefficient	p-value
β0	19.198	0.0435
β1	−2.554	0.0167
β2	0.326	0.0419
β12	−0.087	0.0159
β11	1.222	0.0392
β22	−0.001	0.0545
**Model diagnostic**		
R^2	0.654	
Adj-R^2	0.575	

The statistical analysis confirms that both independent variables X1 and X2 (type of solvent and solvent concentration, respectively) exhibited a significant effect on total phenols (Y1) extraction, with both coefficients (β1 and β2) characterized by a p-value < 0.05. In addition, also the interaction coefficient resulted to have a significant effect

on the re-sponse variable ($p < 0.05$). Finally, the values obtained for R2 and Adj-R2 (0.654 and 0.575, respectively) suggest that the regression model was suitable to describe the observed ex-perimental data.

S3. Biological properties of liquors

The reducing activity of TP and HPH-TP extracted with optimized solvent (acetone at 80% v/v under agitation at 180 rpm at 25°C for 24 h) has been reported in the following table (Table S2) within the reducing activity of liquors out coming from hydrolysis steps. The drying processes, air drying at 50 °C for TP and freeze-drying for HPH pretreated TP, do not affect the reducing activity. Therefore, the initial TPC and FRAP values are not significant ($p < 0.05$) different and the change in biological activity of the liquors do not depends on drying process of the raw materials (TP and HPH-TP) used for the mild se-quential chemical hydrolysis.

Table S2. Reducing activity of TP and HPH-TP liquors coming from solvent extraction and hydrolysis steps.

	**TP**	**HPH-TP**
Solvent extraction	0.29 ± 0.01[d]	0.30 ± 0.01[d]
Acid hydrolysis	0.56 ± 0.02[e]	0.58 ± 0.01[e]
Alkaline hydrolysis	0.10 ± 0.01[b]	0.20 ± 0.02[c]
Bleaching	0.04 ± 0.01[a]	0.05 ± 0.01[a]

Different letters denote statistically significant ($p < 0.05$) differences.

References

1. Sambo F; Borrotti M; Mylona K A Coordinate-Exchange Two-Phase Local Search Algorithm for the D- and I-Optimal Designs of Split-Plot Experiments. *Comput. Stat. Data Anal* **2014**, *71*, 1193–1207, doi:10.1016/j.csda.2013.03.015
2. Myers Raymond H., Montgomery Douglas C., A.-C. CM Response Surface Methodology: Process and Product Optimization Using Designed Experiments; Edition, 4th, Ed.; Wiley: New York, **2016 .**
